# Computational Investigation of Novel pUL56 Ligands Using Docking and Molecular Dynamics with Preliminary Cytotoxicity Evaluation: An Early-Stage Study

**DOI:** 10.3390/molecules31081310

**Published:** 2026-04-17

**Authors:** Viktoria Feoktistova, Samson Olusegun Afolabi, Artem M. Klabukov, Anna A. Shtro, Aleksei V. Kolobov, Ruslan I. Baichurin, Ekaterina V. Skorb, Sergey Shityakov

**Affiliations:** 1 Infochemistry Scientific Center, Information Technologies, Mechanics and Optics (ITMO) University, Saint Petersburg 191002, Russia; feovika0@gmail.com (V.F.); olusegunafolabi2@gmail.com (S.O.A.); kolobovav@ystu.ru (A.V.K.); skorb@itmo.ru (E.V.S.); 2 Smorodintsev Research Institute of Influenza, 15/17 Prof. Popov Str., Saint Petersburg 197376, Russia; temaklab@gmail.com (A.M.K.); anna.shtro@influenza.spb.ru (A.A.S.); 3 Organic and Analytical Chemistry, Yaroslavl State Technical University, 88 Moskovskiy Ave., Yaroslavl 150023, Russia; 4 Organic Chemistry, Herzen State Pedagogical University of Russia, Saint Petersburg 191186, Russia; ruslanio85@mail.ru; 5 Biology and General Genetics, Sechenov First Moscow State Medical University, Moscow 119992, Russia

**Keywords:** human cytomegalovirus, terminase inhibitor, letermovir analog, molecular docking, cytotoxicity, molecular dynamics

## Abstract

Human cytomegalovirus (HCMV) remains a significant cause of morbidity in immunocompromised patients, necessitating the development of improved antivirals. Using an integrated in silico and in vitro approach, we identified a novel ligand (NL) as a letermovir analog with enhanced binding affinity and reduced cytotoxicity. A pUL56 terminase subunit model generated with AlphaFold 3 was used for the virtual screening of a 15,000-compound library. Among the 73 candidates with structural similarity to letermovir (Tanimoto ≥ 0.6), NL exhibited superior predicted binding affinity (ΔG_bind_ = −10.7 kcal/mol). In silico toxicity prediction (ProTox 3.0) classified NL as having low toxicity (class 4, LD50 ≈ 1000 mg/kg), which was confirmed in vitro, where NL demonstrated 158-fold less toxic (CC50 = 2.69 mg/mL) in MRC-5 cells than letermovir (0.017 mg/mL). Molecular dynamics simulations over 500 ns revealed that the pUL56-NL complex forms a more thermodynamically stable interaction, with a lower calculated free energy of binding (MMGBSA: −40.89 ± 7.40 kcal/mol vs. −32.76 ± 4.96 kcal/mol) and a narrower free energy landscape. These results establish NL as a promising, low-cytotoxicity candidate with enhanced target engagement, warranting further investigation as a potential anti-HCMV therapeutic.

## 1. Introduction

HCMV represents a significant public health concern, with the seroprevalence reaching approximately 60% in developed countries and over 90% in developing regions [1]. In immunocompetent individuals, infection is typically asymptomatic because of effective immune control. However, in immunocompromised individuals, HCMV can cause severe disease, affecting multiple organs and tissues and presenting with a broad spectrum of clinical manifestations.

HCMV infection is characterized by lifelong latency and the potential for reactivation [2] and remains a leading cause of morbidity and mortality among bone marrow and solid organ transplant recipients [3], as well as other immunocompromised populations [4]. The clinical manifestations of HCMV can include mononucleosis-like syndrome, headache, malaise, fever, and lymphocytosis, whereas less common presentations include anemia, splenomegaly, and exudative pharyngitis [5]. Furthermore, HCMV is a major cause of congenital infections, is transmissible from mother to fetus, and represents a leading infectious contributor to deafness and neurodevelopmental disabilities in children [6].

However, no vaccines are approved for the prevention or treatment of HCMV infections. Moreover, despite its clinical significance, therapeutic options for HCMV are limited. Several targets are currently being considered in the context of developing new drugs. One of the most promising of these targets is the viral terminase complex, which is responsible for processing replicated viral DNA. During replication, HCMV generates concatemers in which multiple genome copies are joined in a head-to-tail arrangement. The terminase complex cleaves these concatemers into unit-length genomes that are subsequently packaged into capsids [7]. In HCMV, the terminase complex is composed primarily of two subunits: the ATPase pUL56 and the nuclease pUL89 [8], with pUL51 [9] sometimes contributing as an additional component.

Letermovir (LET) is the first antiviral agent whose mechanism of action is based on the inhibition of the viral terminase complex [10]. This drug is thought to exert its antiviral activity by binding to the pUL56 subunit [11]. The precise mechanism of action of pUL56 remains to be fully elucidated, and there is not much information about pUL56 and the whole terminase complex in the literature. Evidence supporting pUL56 as the primary target comes from observations that resistance to LET is predominantly associated with mutations in pUL56, rather than in the other subunits of the complex [12], and that certain mutations in this protein influence the degree of viral resistance to LET [13]. This subunit has a particular structure with a pronounced cleft and is capable of binding to DNA and associating with the capsid, but pUL89 is not responsible for cutting DNA [14]. A conserved sequence is known to form the region responsible for the interaction of pUL56 and pUL89 [15].

Subsequent studies have demonstrated that LET is generally well tolerated and highly effective [16] and is used both for prophylaxis and treatment of HCMV infection [17], including in the context of organ transplantation, to reduce patient morbidity and mortality [18]. A key advantage of LET is its selectivity for the viral terminase complex, which has no human homolog [19], ensuring targeted antiviral activity. Nevertheless, treatment with LET may be associated with adverse events such as gastroenteritis, nasopharyngitis, dyspnea, and elevated serum creatinine [20]. According to the official Merck website, the most frequently reported side effects include nausea, diarrhea, vomiting, peripheral edema, cough, headache, fatigue, and abdominal pain [21].

**Figure 1:**
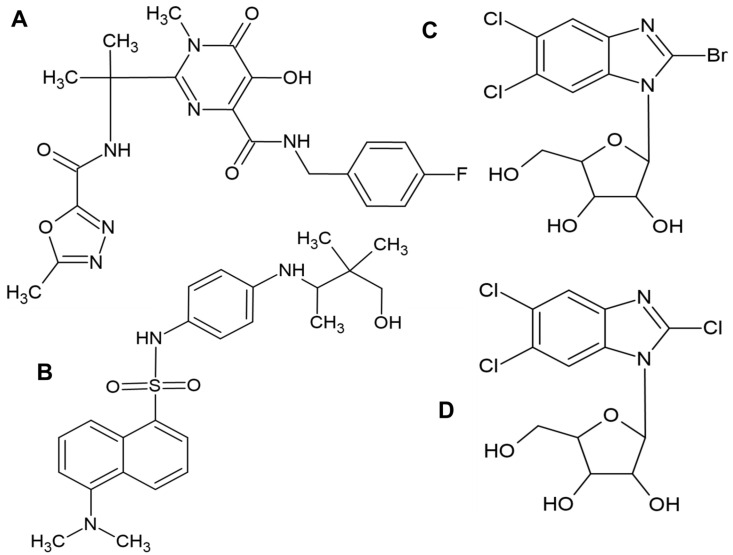
Structures of HCMV terminase-targeting compounds investigated as potential therapeutics. (**A**) Raltegravir; (**B**) Tomeglovir; (**C**) 2-bromo-5,6-dichloro-1-(β-D-ribofuranosyl)benzimidazole; (**D**) 2,5,6-trichloro-1-β-D-ribofuranosylbenzimidazole. These compounds have been studied as potential inhibitors but are not used clinically.

In the literature, few attempts have been made to identify compounds capable of acting via a mechanism similar to LET; however, no significant progress has been made. One candidate, tomeglovir (Bay38-4766), is a chemically unrelated terminase complex inhibitor. This compound has some efficacy in animals but is capable of inducing strong resistance in viruses. Tomeglovir can serve as a prototype and can be used for research purposes but is not registered for clinical use [22]. pUL56 interacts with the viral portal protein pUL104 during DNA packaging through the C-terminal part of pUL56, which is important for DNA packaging, and this process can be interrupted by 2-bromo-5,6-dichloro-1-(β-D-ribofuranosyl)benzimidazole and 2,5,6-trichloro-1-beta-D-ribofuranosylbenzimidazole [23]. Raltegravir derivatives have been proposed to inhibit pUL89, another HCMV terminase subunit. These compounds (Figure 1A–D) have been tested in silico and in vitro, but no information on follow-up experiments has been found [24]. Collectively, these data indicate that the terminase complex remains a promising but insufficiently explored antiviral target, and the lack of structural information limits rational ligand design.

**Figure 2:**
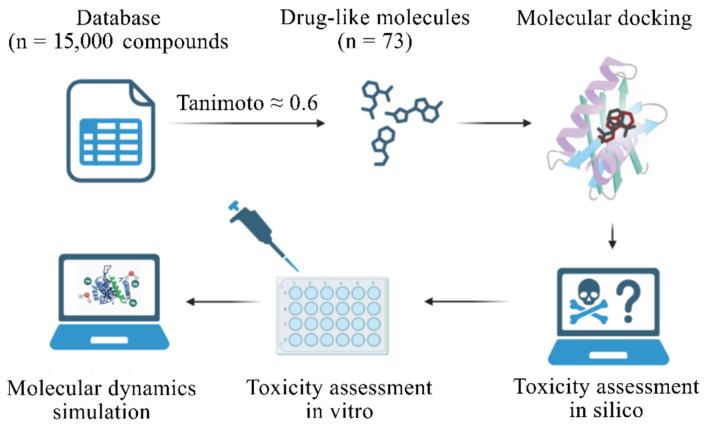
The workflow of the NL search. First, a database containing 15,000 compounds was selected to identify those with a similarity to LET of at least 0.6 according to the Tanimoto coefficient. The selected structures were analyzed via molecular docking. The toxicity of the compound with the best results was assessed in silico. An MTT assay was then conducted to compare the CC_50_ values of LET and NL. Finally, molecular dynamics simulations were conducted on the pUL56-LET and pUL56-NL complexes.

The aim of this study was to identify promising inhibitors of pUL56 and thereby accelerate the discovery of potential anti-CMV compounds. Since the experimentally determined structure of pUL56 was not available at the time of the work, a predicted model obtained via AlphaFold3 [25] was used. This strategy is widely applied in modern research not only in the absence of experimental structures but also for analyzing mutation effects [26,27,28]. AlphaFold3 has been shown to accurately reproduce the global architecture of the receptor and orthosteric binding pockets, as demonstrated for 74 G protein-coupled receptor structures [29], supporting its applicability for structure-based drug discovery. A novel potential ligand (NL) was identified during the virtual screening of a 15,000-compound library, and its toxicity was evaluated through both in silico and in vitro approaches. The substance demonstrated higher binding efficiency to pUL56 and lower toxicity in the experiments conducted. The overall workflow of the study is presented in Figure 2.

## 2. Results and Discussion

### 2.1. Compound Database

**Figure 3:**
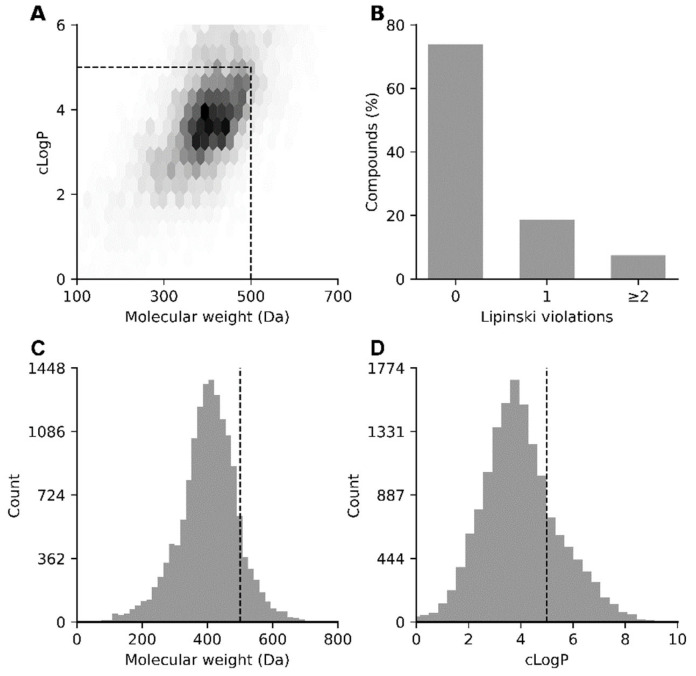
Lipinski analysis of the Globus database. (**A**) Two-dimensional density plot of the molecular weight versus the calculated octanol–water partition coefficient (cLogP). The dashed rectangle indicates the canonical drug-like region defined by Lipinski’s rule of five. (**B**) Distribution of compounds according to the number of Lipinski rule-of-five violations. (**C**) Histogram of molecular weight values with a Lipinski threshold of 500 Da indicated by a dashed vertical line. (**D**) Histogram of cLogP values with a Lipinski threshold of 5 indicated by a dashed vertical line.

To assess the drug likeness of the compounds in the database used, key molecular descriptors were analyzed in accordance with Lipinski’s rule of five. The distributions of the molecular weight versus clogP and the number of Lipinski violations are summarized in Figure 3A–D.

Like any other rule-based approach, Lipinski’s rule of five defines specific ranges of values for a number of parameters, and a molecule that fits within these ranges is considered drug-like. This approach is convenient because of its ease of interpretation, but there is a risk of overnarrowing the chemical space.

The majority of molecules are located within the drug-like region (molecular weight ≤ 500 Da, clogP ≤ 5), indicating that the library is largely compliant with Lipinski’s criteria; however, a fraction of compounds that are not included in this region can also be considered potential drug-like substances. Many FDA-approved drugs do not comply with this rule [30]. Moreover, antiviral drugs often have a more complex structure than other drug classes do and violate one or more of Lipinski’s rules, especially when they involve prodrugs or nucleotides. This can often be compensated for by a special route of administration or metabolic activation [30]. For example, remdesivir is a nucleotide analog administered intravenously to treat severe forms of COVID-19. Its molecular weight is 602.6 g/mol (exceeding the 500 D limit established by the Lipinski rule), and it is converted into active triphosphate in cells [31].

### 2.2. Compound Structure and Purity Confirmation

**Figure 4:**
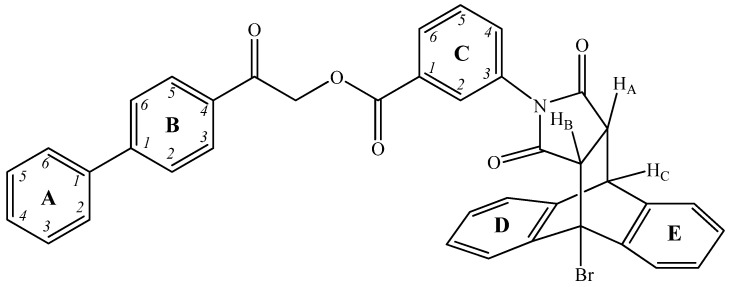
Structure of NL with rings, key atoms and protons labeled for NMR.

^1^H NMR: 3.58 (1H, dd, ^3^*J*_AB_ 8.5, ^3^*J*_AC_ 3.2 Hz, H_A_), 3.66 (1H, d, ^3^*J*_AB_ 8.5, H_B_), 4.58 (1H, d, 3.2 Hz, H_C_), 5.79 (2H, s, OCH_2_), 6.80 (1H, ddd, ^3^*J*_3-4_ 7.9, ^4^*J*_3-5_ 1.1, ^4^*J*_3-2_ 1.8 Hz, H^3^, Ar_C_), 7.11 (1H, t, <^4^*J*> 1.8 Hz, H^2^, Ar_C_), 7.27–7.34 (4H, m, Ar_D,E_), 7.34–7.38 (1H, m, Ar_D,E_), 7.42 (1H, t, <^3^*J*> 7.5 Hz, H^4^, Ar_A_), 7.50 (2H, t, <^3^*J*> 7.8 Hz, H^3,5^, Ar_A_), 7.50 (2H, t, <^3^*J*> 7.8 Hz, H^3,5^, Ar_A_), 7.56 (1H, t, <^3^*J*> 7.9 Hz, H^4^, Ar_C_), 7.57–7.59 (1H, m, Ar_D,E_), 7.60–7.64 (1H, m, Ar_D,E_), 7.75 (2H, d, <^3^*J*> 7.9 Hz, H^2,6^, Ar_A_), 7.79–7.83 (1H, m, Ar_D,E_), 7.87 (2H, d, ^3^*J* 8.4 Hz, H^2,6^, Ar_B_), 7.98 (1H, dt, ^3^*J* 7.9 Hz, <^4^J> 1.6, H^5^, Ar_C_), 8.08 (2H, d, ^3^*J* 8.4 Hz, H^3,5^, Ar_B_). ^13^C{^1^H} NMR: 44.94 (CH_C_), 49.46 (CH_A_), 54.15 (CH_B_), 65.93 (CBr), 68.01 (OCH_2_), 124.70 (Ar), 125.25 (Ar), 125.37 (Ar), 125.65 (Ar), 127.47 (Ar), 127.62 (C^2,6^, Ar_A_), 127.66 (C^2,6^, Ar_B_), 127.90 (Ar), 128.21 (C^2^, Ar_C_), 128.42 (Ar), 128.68, 129.13 (C^4^, Ar_A_), 129.16 (C^3,5^, Ar_B_), 129.68 C^3,5^, Ar_A_, 130.05 (Ar), 130.33 C^5^, Ar_C_), 130.63 (C^4^, Ar_C_), 132.21 (C^3^, Ar_C_), 132.65 (Ar), 133.15 (C^4^, Ar_B_), 138.16 (Ar), 139.00 (Ar), 139.27 (C^1^, Ar_A_), 140.76 (Ar), 141.27 (Ar), 145.92 (C^1^, Ar_B_), 164.77 [C(O)O], 172.97 [C(O)N], 174.92 [C(O)N], 192.57 [C(O)Ar] (Figure 4; Supplementary Figures S2–S7).

The mass spectrometry and infrared spectroscopy data further corroborate the proposed structure and confirm the purity of the compound, complementing the detailed NMR analysis. High-resolution mass spectrometry (HRMS) analysis via electrospray ionization in positive mode (ESI^+^) revealed a prominent ion peak at *m*/*z* 690.0891 corresponding to the sodium adduct [M + Na]^+^. The observed mass is in excellent agreement with the calculated value for C_39_H_26_BrNO_5_Na (calcd. 690.0900). The isotopic pattern shows a characteristic 1:1 doublet separated by 2 Da, confirming the presence of a single bromine atom in the molecule (Supplementary Figure S8). The IR spectrum shows characteristic carbonyl bands, which further confirms the structure of the analyzed substance (Supplementary Figure S9).

### 2.3. Creating a Protein Model

As noted previously, several promising targets have been identified that are potentially useful for anti-HCMV drug development. The DNA polymerase UL54 [32] is a classic target. Among the drugs that target this protein are ganciclovir and its prodrugs valganciclovir [33], foscarnet [34], and cidofovir [35]. This target remains important, but the toxicity of drugs that target it and the frequent emergence of resistance stimulate the search for new targets. Second, the kinase UL97 [36] is the target of maribavir. Maribavir functions by inhibiting the viral protein kinase pUL97, thereby interfering with viral morphogenesis and the release of newly formed virions from the nucleus. However, pUL97 is also required for the initial phosphorylation of ganciclovir, resulting in pharmacological antagonism when maribavir and ganciclovir are used concomitantly [37]. Although maribavir is associated with a lower incidence of severe adverse effects [38], its clinical use is limited because of its narrow antiviral spectrum, its activity exclusively against HCMV [37], and its poor compatibility with other antiviral agents.

A potential binding site for LET in the pUL56 subunit has recently been identified [39]. Briefly, structure prediction was performed via the Phyre2 web portal [40] and then optimized via the H++ server [41]. The potential binding site was identified with SwissDock [42]. The resulting structure was highly homologous to the crystal structure of the small subunit of herpes simplex virus type 1 (HSV-1; strain 17) terminase pUL28 (PDB: 6m5u, B-chain) and to that of human topoisomerases (homology confidence 85–90%). A BLAST search via KEGG (https://www.genome.jp/kegg/; accessed on 15 August 2025) for the pUL56 sequence revealed the following pdbstr sequences: 6M5SB, 6M5UB, 6M5RB, 6M5VB, 5HUYB, and 5HUYA. A BLAST search in NCBI provides 6M5RB and 6M5VB, the query cover for which is the same: 83%. It is believed that resistance to LET occurs through mutations that result in changes in the shape and size of the pocket and subsequent expulsion of the LET molecule from the terminase complex. The pUL56 model obtained in this work is consistent with the data from the literature [39,43].

### 2.4. Molecular Docking

There were 73 substances whose similarity to LET according to the Tanimoto coefficient was ≈0.6 (Supplementary Table S1). A substance is considered to bind effectively to a receptor if the affinity value is −6.0 kcal/mol or less [44]. pUL56—LET had ΔG_bind_ = −8.7 kcal/mol in AutoDock vina and ΔG_bind_ = −7.75 kcal/mol in AutoDock 4.2.6. On the basis of the docking results, the most promising ligand (s61, hereinafter referred to as NL) was determined to have ΔG_bind_ = −10.1 kcal/mol in AutoDock Vina and ΔG_bind_ = −10.7 kcal/mol in AutoDock 4.2.6.

**Figure 5:**
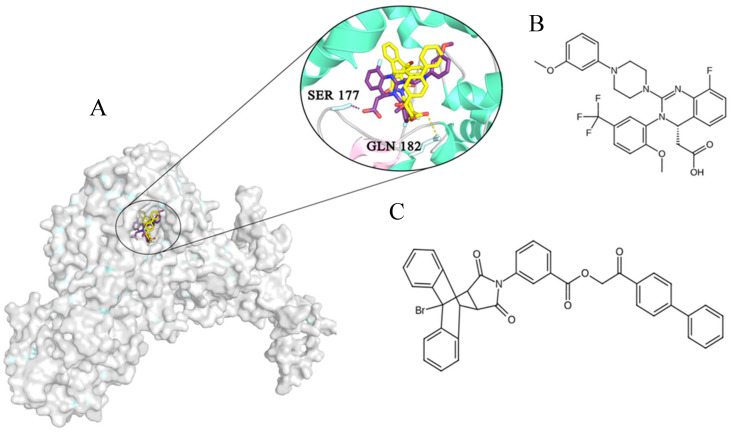
3D structure (**A**) of pUL56 generated with AlphaFold 3 with the binding site and ligands: (**B**) LET (shown in violet) and (**C**) NL (shown in yellow).

Both AutoDock Vina and AutoDock 4.2.6 are widely used classic tools for assessing the Gibbs free energy of binding. However, AutoDock Vina is better at predicting ligand poses, whereas AutoDock 4 is more accurate at determining binding energies [45]. The formulas of LET and NL and their positions in the binding site of pUL56 are presented in Figure 5A–C.

### 2.5. In Silico Toxicity Analysis

As mentioned before, ProTox 3.0 is a toxicity prediction web server that employs an integrated modeling framework. All the models used for toxicity prediction were validated via cross-validations and external validation sets [46]. However, the predictions remain probabilistic and limited by the applicability domain. Moreover, toxicity effects can be shown not only by the drug itself but also by its metabolically generated intermediates, the prediction of which is more complicated [47].

According to ProTox 3.0 (Table 1), both LET and NL are class 4 toxins, but the LD_50_ values differ significantly in favor of NL (500 mg/kg for LET and 1000 mg/kg for NL). However, in nonclinical reviews [48], LET has a greater safety profile.

The manufacturer claims that the acute oral toxicity of LET is more than 5000 mg/kg. The no observed adverse effect level (NOAEL) for rats (14-day bolus administration) is 30 mg/kg/day because of adverse liver and male reproductive system findings. When administered by gavage for 4 weeks, the NOAEL in rats is 60 mg/kg/day due to adverse findings in the liver (increased liver weight, histopathology findings, variations in liver enzyme activity, and clinical chemistry and urinalysis findings) and male reproductive system (histopathology findings) at the high dose. The lowest observed adverse effect level (LOAEL) for liver effects in rats in a 4-week study was 9.8; in a 13-week study, it was 8.1; and the LOAEL for testicular effects in rats in a 13-week study was 8.1. Impaired spermatogenesis/vacuolization of the epithelium in the seminiferous tubules and decreased fertility at doses well above clinical exposure in rats at relatively high exposure levels. The FDA noted the risk to the male reproductive system. In studies in rabbits and rats at doses several times greater than the clinical exposure, changes in fetal development and/or reduction in fetal size were observed. LET does not exhibit genotoxic or mutagenic properties in standard in vitro or in vivo tests. In the clinical phase, no pronounced hematotoxicity (neuropenia/leukopenia) was detected, and no pronounced nephrotoxicity was detected, which distinguishes LET favorably from classical drugs such as ganciclovir and foscarnet. The most common side effects are nausea, diarrhea, edema, and, less commonly, increased liver enzymes. Severe side effects, such as atrial fibrillation or flutter, increased alanine aminotransferase levels, and hyperkalemia, have rarely been observed [49]. Isolated cases of transaminitis [50], hepatotoxicity and lactic acidosis have been reported [48].

### 2.6. In Vitro Toxicity Analysis

**Figure 6:**
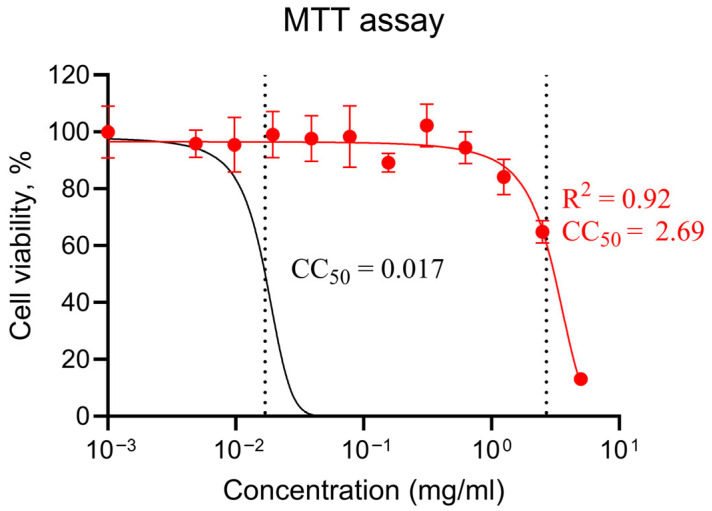
MTT cytotoxicity of LET and NL. Dose-response curve for NL and calculated CC_50_ for LET. The thresholds are depicted as dotted lines.

In this work, MRC5 cells were subjected to the MTT test. This cell type was chosen on the basis of several articles testing LET, the reference compound, specifically in fibroblasts [10,51,52]. NL had a CC_50_ = 2.69 mg/mL, whereas for LET under the same conditions, a CC_50_ ≈ 0.017 mg/mL was recorded (Figure 6). In terms of mass, this corresponds to an approximately 158-fold decrease in NL cytotoxicity compared with that of LET in this study (Figure 5). However, without data on antiviral activity (EC_50_) and in the absence of confirmation in additional cytotoxic tests (ATP, LDH) and under the same experimental conditions, such a comparison should be considered preliminary.

### 2.7. Molecular Dynamics Simulation

**Figure 7:**
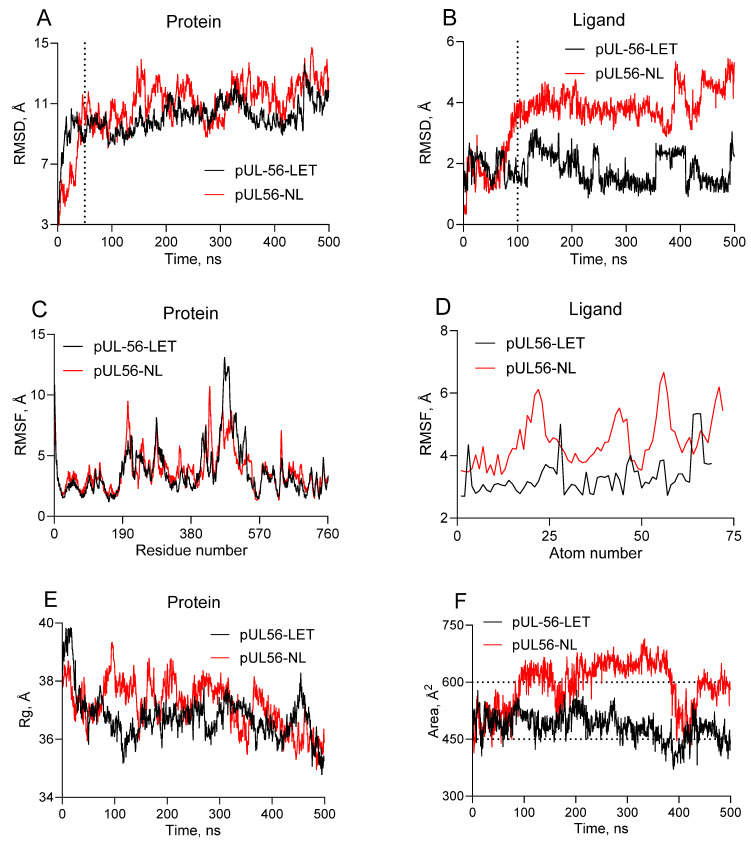
Molecular dynamics results for pUL56-LET (black) and pUL56-NL (red). (**A**)—Root mean square deviation of Cα atoms of protein; (**B**)—Root mean square deviation of ligands; (**C**)—Root mean square fluctuation of protein; (**D**)—Root mean square fluctuation of ligand atoms; (**E**)—Radius of gyration of protein; (**F**)—Buried surface area (BSA). The thresholds are shown as dotted lines.

Once the toxicity of the compounds was determined, MD simulations of the complexes were carried out. The RMSD values (Figure 7A,B) revealed that the pUL56-LET complex exhibited faster constant structural behavior than did the pUL56-NL complex. The RMSD of pUL56 in complex with LET remained relatively stable throughout the simulation, with a mean value of 10.31 ± 0.90 Å, indicating limited structural deviation. In comparison, the RMSD for the pUL56-NL complex was greater, averaging 11.43 ± 1.16 Å, suggesting greater flexibility in this complex. Ligand binding does not significantly alter protein flexibility (Figure 7C). NL was more flexible than letermovir, but this did not interfere with the efficiency of interaction with the protein (Figure 7D). By assessing the changes in the radius of gyration, it can be assumed that both complexes exhibit similar behavior (Figure 7E). Comparative MD analysis of pUL56 complexes with LET and NL may indicate the formation of “stable” complexes throughout the 500 ns simulation. Buried surface (BSA) analysis (Figure 7F) revealed that both ligands maintain contact with pUL56, but the contact area of NL with the protein is larger than that of LET, which may indicate more stable binding.

Furthermore, the free energies of binding among the ligands and protein were estimated via the MMGBSA and MMPBSA approaches (Table 2). For the LET complex, the calculated energies were −32.76 ± 4.96 kcal/mol for MMGBSA and −60.19 ± 6.58 kcal/mol for MMPBSA. The NL complex energies were −40.89 ± 7.40 kcal/mol and −76.53 ± 10.99 kcal/mol, respectively. Both MMGBSA and MMPBSA calculations consistently indicate that NL displays a stronger binding affinity toward the protein than LET does, as reflected by more negative ΔG_bind_ values.

**Figure 8:**
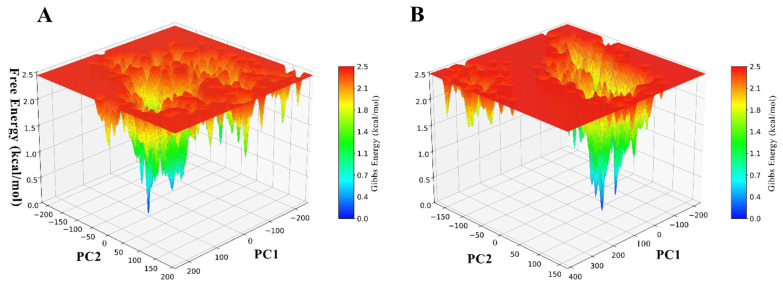
Three-dimensional FELs of protein-ligand complexes projected onto the first two principal components (PC1 and PC2). (**A**)—FEL of the pUL56–LET complex; (**B**)—FEL of the pUL56–NL complex.

Compared with the pUL56-LET complex, the pUL56-NL complex has superior protein-ligand interactions, as evidenced by its free energy landscape characteristics (Figure 8A,B). It exhibits fewer distinct meta-stable states, with the low-energy basins appearing more consolidated and less fragmented, indicating that the ligand stabilizes a narrower, more focused ensemble of conformations. The energy funnel in pUL56–NL is noticeably narrower, reflecting a steeper and more directed pathway toward the global minimum (the bound state), which suggests more efficient conformational selection or induced fit. Additionally, the high-energy meta-states and surrounding barriers are less spread out and more confined, implying that unproductive or off-pathway conformations are suppressed, resulting in a higher effective energy threshold for escaping the bound basin. Together, these features point to stronger thermodynamic stabilization, reduced conformational entropy in the bound state, and likely an increased dissociation barrier, all of which are hallmarks of tighter binding affinity and potentially longer residence time, making the ligand in pUL56–NL the better binder overall.

## 3. Materials and Methods

### 3.1. Synthesis of the Selected Hit Compound

The novel ligand (NL) as letermovir analog (Synthesis of 3-(4-Bromo-1,3,3a,4,9,9a-hexahydro-1,3-dioxo-4,9[1′,2′]-benzeno-2H-benz[f]isoindol-2-yl)benzoic acid (CAS 332031-41-1)) was prepared according to the general procedure for imide formation developed by our team. A mixture of 9-bromo-9,10,11,15-tetrahydro-9,10[3′,4′]-furanoanthracene-12,14-dione (CAS 58802-01-0; 0.005 mol), m-aminobenzoic acid (0.005 mol), and glacial acetic acid (15 mL) was heated under reflux for 2 h. Upon cooling to room temperature, the reaction mixture was poured into a beaker and allowed to stand overnight. The precipitated solid was collected by filtration, washed with cold acetic acid, and dried under vacuum to afford the product in 85–95% yield. Phenacyl ester formation was carried out following the general protocol. To a solution of the above benzoic acid derivative (0.00553 mol) and triethylamine (0.9 mL) in acetone (30 mL) or a 1:1 (*v*/*v*) mixture of acetone and DMF when required for solubility, 1-([1,1′-biphenyl]-4-yl)-2-bromoethanone (CAS 135-73-9; 0.0057 mol) was added. The reaction mixture was heated to reflux with stirring for 2 h. After cooling, the mixture was poured into water (100 mL), and the resulting precipitate was collected by filtration. The crude product was recrystallized from ethanol (25 mL) to yield pure phenacyl ester in 77–97% yield (Supplementary Figure S1). The compound structure and purity were confirmed via NMR spectroscopy, IR spectroscopy and mass spectrometry.

^1^Н, ^13^С NMR spectra, ^1^H–^13^C HMQC, ^1^H–^13^C HMBC, ^1^H−^1^H dqf-COSY and NOESY (mixing time from 0.5 to 2 s) experiments were acquired on a Jeol ECX400A spectrometer (Akishima, Japan, 400 MHz for ^1^H nuclei and 100 MHz for ^13^С nuclei) in DMSO-*d*_6_. The residual signals of the solvent (DMSO-*d*_6_: 2.50 ppm for ^1^Н nuclei and 39.6 ppm for ^13^С nuclei) were used as internal standards. Vibrational spectra were recorded on a Shimadzu IRPrestige-21 IR Fourier spectrometer in KBr.

Spectral studies were performed using the equipment of the Center for Collective Use «Physicochemical methods for the study of nitro compounds, coordination compounds, biologically active substances, and nanostructured materials» of the Interdisciplinary Resource Center for Collective Use «Modern physicochemical methods of formation and research of materials for the needs of industry, science, and education» of the Herzen State Pedagogical University of Russia.

Mass spectra were obtained via electrospray ionization (ESI) in positive mode on a Bruker micrOTOF time-of-flight mass spectrometer (Billerica, MA, USA). Ions were recorded in the *m*/*z* range of 50–1300. The capillary voltage was 4500 V, the plate bias was −500 V, the nebulizer pressure was 0.4 bar, the drying gas temperature was 180 °C, and the gas flow rate was 4.0 L/min. The measurements were carried out using the equipment of the Research Park of St. Petersburg State University.

### 3.2. Database Filtering

The proprietary compound database comprises approximately 15,000 compounds in SDF format. The molecular descriptors of the compounds and their compliance with Lipinski’s rule of five were analyzed via the Python libraries pandas (Version: 2.3.2), NumPy (Version: 1.26.4) and Matplotlib (Version: 3.10.1). Lipinski’s rule of five helps identify molecules that meet a set of criteria common to most successful drug molecules. This rule remains popular and is often used in medical research [53,54,55].

From this collection, molecules with a Tanimoto similarity coefficient ≥ 0.6 relative to LET were selected. The threshold was intentionally lowered from the commonly used value of 0.7 to increase the number of candidate compounds [56,57,58]. The selected compounds were converted to PDB format via Open Babel software (Version: 3.1.1).

The Tanimoto similarity score is one of the most commonly used similarity metrics. It is calculated as the ratio of the total number of common features to the total number of unique features, taking values from 0 to 1, where 0 means no similarity and 1 means a complete match. The Tanimoto coefficient is considered one of the best for assessing molecular similarity, which has been shown in several studies [59,60].

### 3.3. Protein Model Design

The amino acid sequence of HCMV pUL56 was sourced from the NCBI database (ProteinID: YP_081515). This sequence was used to create a model of pUL56 via the AlphaFold3 pipeline. The resulting model was processed via an in-house script (unpublished) via the SWISS-MODEL platform (https://swissmodel.expasy.org/; accessed on 18 June 2025). The structure obtained after loop refinement was analyzed via the CASTp 3.0 server (http://sts.bioe.uic.edu/castp/; accessed on 18 June 2025), where the binding site was found. Its coordinates were determined to be as follows: grid box center: x = 16.02 Å; y = −5.94 Å; z = −23.88 Å.

### 3.4. Two-Phase Molecular Docking

Molecular docking was performed in two phases: first, virtual screening was performed via AutoDock Vina (Version 1.1.2) with standard parameters [44,61], where the grid box size in the x, y and z dimensions = 22.50 Å as the default dock box volume [62,63]. In the second phase, the molecules that showed the best affinity results were analyzed via AutoDock 4.2.6 with a grid box size of 60 Å in all dimensions [44,64].

### 3.5. In Silico Toxicity Analysis

ProTox 3.0 (https://tox.charite.de/protox3/; accessed on 10 September 2025) is a toxicity prediction web server that employs an integrated modeling framework that combines multiple computational strategies, including molecular similarity analysis, fragment-based molecular descriptors, pharmacophore-derived features, and machine learning–based predictive models [65]. The predictive models are implemented in Python via the machine learning library scikit-learn [66] and the cheminformatics toolkit RDKit [67] for molecular representation and descriptor calculation. Data preprocessing, standardization and workflow management were performed via the KNIME Analytics Platform [68], a workflow-based environment for reproducible data analysis and the integration of computational tools.

To ensure the robustness and generalizability of its predictive models, ProTox 3.0 applies both internal validation procedures—typically k-fold cross-validation—and external validation using independent test sets. This dual-level evaluation strategy strengthens confidence in the reproducibility, stability, and real-world applicability of the toxicity predictions [46].

### 3.6. MTT Assay

MRC-5 cell cultures (human lung fibroblasts) were obtained from the working collection of cell cultures of the Laboratory of Vector Vaccines of the Federal State Budgetary Institution “Smorodintsev Research Institute of Influenza”. To determine the cytotoxic effect of the drug-like molecule, a series of twofold dilutions were prepared starting from 1/2. A 24 h cell culture grown in 96-well plates at a concentration of 3 × 10^5^ cells/well was visually inspected for monolayer integrity under a P2-1 inverted biological microscope (Biolam, LOMO, Saint Petersburg, Russia). The dilutions of the preparations at the appropriate concentration (from 5 mg/mL to 0.002 mg/mL) were added to the plates at a volume of 100 μL in each well, with 8 parallels for each tested concentration in 3 replicates. A row of cell controls was used as a negative control. The plates with dilutions of the preparations were incubated for 7 days at 37 °C in the presence of 5% CO_2_ in a CO_2_ incubator MCO-18 (Sanyo, Daito-shi, Japan). The MTT assay was conducted via the classical method. After 2 h of contact, the MTT reagent was discarded, and 0.1 mL of DMSO was added, after which the optical density in the wells was measured on an AMR-100 microplate reader (Allsheng, Hangzhou, China) at a wavelength of 570 nm. The cytotoxic concentration 50% (CC_50_) was calculated via the GraphPad Prism (Version 10.6.1, academic license) program via asymmetric nonlinear regression analysis.

### 3.7. Molecular Dynamics Simulation

Molecular dynamics simulations were performed via the AMBER 22 package. The ff99SB force field was used for the protein, and the GAFF2 force field was used for the ligand. Partial charges for the ligand were assigned via the Antechamber module via the AM1-BCC method. The system was solvated in a TIP3P explicit water box and neutralized by adding 15 Na^+^ counterions. Production molecular dynamics simulations were performed for 500 ns with a 2-fs timestep in the NVT ensemble. SHAKE constraints were applied to all bonds involving hydrogens, and long-range electrostatics were treated with the particle-mesh Ewald method using a 9 Å nonbonded cutoff. The temperature was maintained at 300 K via a Langevin thermostat [69,70]. Binding affinity was assessed by calculating the Molecular Mechanics Poisson–Boltzmann Surface Area (MM/PBSA) and Molecular Mechanics Generalized Born Surface Area (MM/GBSA) via the AMBER 12 package. The obtained trajectories were analyzed to assess structural stability, flexibility, and conformational dynamics. Specifically, the following analyses were performed: root-mean-square deviation (RMSD) of the protein backbone and ligand, root-mean-square fluctuation (RMSF) of individual residues, radius of gyration (Rg), and buried surface area (BSA) at the protein–ligand interface.

Principal component analysis (PCA) was conducted on the Cα atoms of the protein to extract dominant collective motions. The first two principal components (PC1 and PC2) were then used to construct a two-dimensional free energy landscape (FEL) via Python-based scripts with NumPy (Version: 1.26.4), SciPy (Version: 1.14.0), and Matplotlib (Version: 3.10.1). Topologically relevant low-energy conformations were identified from the FEL. All trajectory processing and calculations were performed via CPPTRAJ and custom scripts, whereas visualization of the structures and graphs was performed via Matplotlib.

## 4. Conclusions

In conclusion, this integrated in silico and in vitro study successfully identified and characterized NL as a highly promising lead candidate for the development of next-generation anti-HCMV therapeutics. The applied pipeline, which combines protein structure prediction, virtual screening, binding energy estimation, cytotoxicity assessment, and molecular dynamics simulations, provides convergent computational evidence supporting a favorable profile of the compound. Within the limits of computational modeling, NL demonstrated greater predicted binding characteristics and thermodynamic stability toward the HCMV terminase subunit pUL56 than the reference compound did, while NL also exhibited substantially lower cytotoxicity (~158-fold) in MRC-5 fibroblasts. The stability of the pUL56–NL complex and its low-energy conformational states observed via molecular dynamics simulations are consistent with the potential for a more durable antiviral effect. These findings collectively establish NL as a promising prototype that addresses key limitations of current terminase inhibitors by prioritizing both target potency and cellular safety. This study is primarily a computational and early preclinical profiling of a novel LET analog, focusing on target engagement and cytotoxicity. The determination of antiviral efficacy (EC_50_) is planned as a critical next step in validation and will be reported in a subsequent publication.

## Figures and Tables

**Table 1:** In Silico toxicity prediction.

Program	Parameter	LET		NL
		Experimental Value	Predicted Value	Predicted Value
ProTox 3.0	Class of toxicity	4	5	4
	Oral rat LD50 mg/kg	>2000 mg/kg	500 mg/kg	1000 mg/kg

**Table 2:** MMPBSA- and MMGBSA-based energy terms for the analyzed substances.

Method	System	Part	Average	Std. Dev	Std. of Mean
MMGBSA	pUL56- LET complex	Complex	−21,649.86	93.87	2.97
		Receptor	−21,542.29	92.79	2.93
		Ligand	−74.80	6.14	0.19
		Difference *	−32.76	4.96	0.16
MMGBSA	pUL56- NL complex	Complex	−21,562.29	88.68	2.80
		Receptor	−21,565.99	86.64	2.74
		Ligand	44.59	5.96	0.19
		Difference *	−40.89	7.40	0.23
MMPBSA	pUL56- LET complex	Complex	−15,094.35	125.26	3.96
		Receptor	−15,009.27	123.99	3.92
		Ligand	−24.89	6.13	0.19
		Difference *	−60.19	6.58	0.21
MMPBSA	pUL56- NL complex	Complex	−15,022.28	119.52	3.78
		Receptor	−15,050.90	117.07	3.70
		Ligand	105.15	6.16	0.19
		Difference *	−76.53	10.99	0.35

* Complex—Receptor—Ligand.

## Data Availability

All the data and analysis scripts have been deposited in the GitHub repository “Letermovir” (https://github.com/VikFeokt/Letermovir (accessed on 14 December 2025)). Docking inputs and outputs, compound databases, and MTT assay results are available. Molecular dynamics input files and representative output data are included; full MD trajectories are available from the corresponding author upon reasonable request.

## Supplementary Materials

The following supporting information can be downloaded at: https://www.mdpi.com/article/10.3390/molecules31081310/s1.

## Abbreviations

**B**SA	buried surface area
**F**DA	Food and Drug Administration.
**F**EL	free energy landscape.
**H**CMV	human cytomegalovirus.
**L**ET	letermovir.
**M**MGBSA	Molecular Mechanics Generalized Born Surface Area.
**M**MPBSA	Molecular Mechanics Poisson-Boltzmann Surface Area.
**N**L	novel letermovir analog.
**P**CA	principal component analysis
**R**g	radius of gyration
**R**MSD	root-mean-square deviation
**R**MSF	root-mean-square fluctuation
