# Supplementary material for: Computational Investigation of Novel pUL56 Ligands Using Docking and Molecular Dynamics with Preliminary Cytotoxicity Evaluation: An Early-Stage Study
Source: Molecules. 2026 Apr 17;31(8):1310. doi: 10.3390/molecules31081310 (PMC13118375; doi:10.3390/molecules31081310)
Supplement: Supplementary file 1 [file molecules-31-01310-s001.zip › molecules-4160395-supplementary.pdf]

Supplementary Material

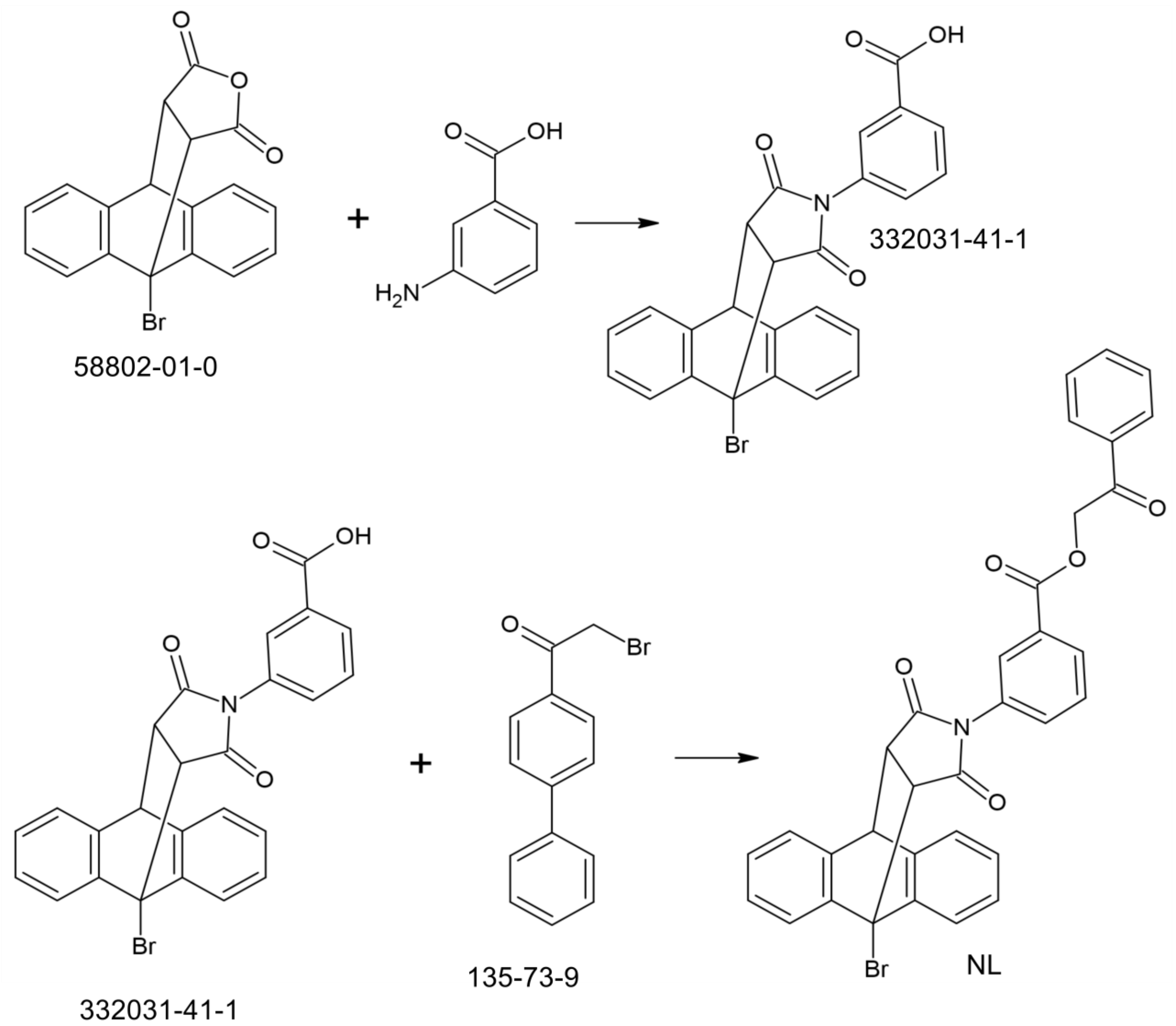

Figure S1. The scheme shows the final product obtained after a modified Williamson ester synthesis of the imide-containing benzoic acid with the phenacyl bromide reagent. Chemical structure of the phenacyl ester derivative: 3-(4-Bromo-1,3,3a,4,9,9a-hexahydro-1,3-dioxo-4,9[1′,2′]-benzeno-2H-benz[f]isoindol-2-yl)benzoic acid 1-([1,1′-biphenyl]-4-yl)-2-oxoethyl ester (or the corresponding phenacyl ester formed by reaction with 1-([1,1′-biphenyl]-4-yl)-2-bromoethanone).

NMR Spectra

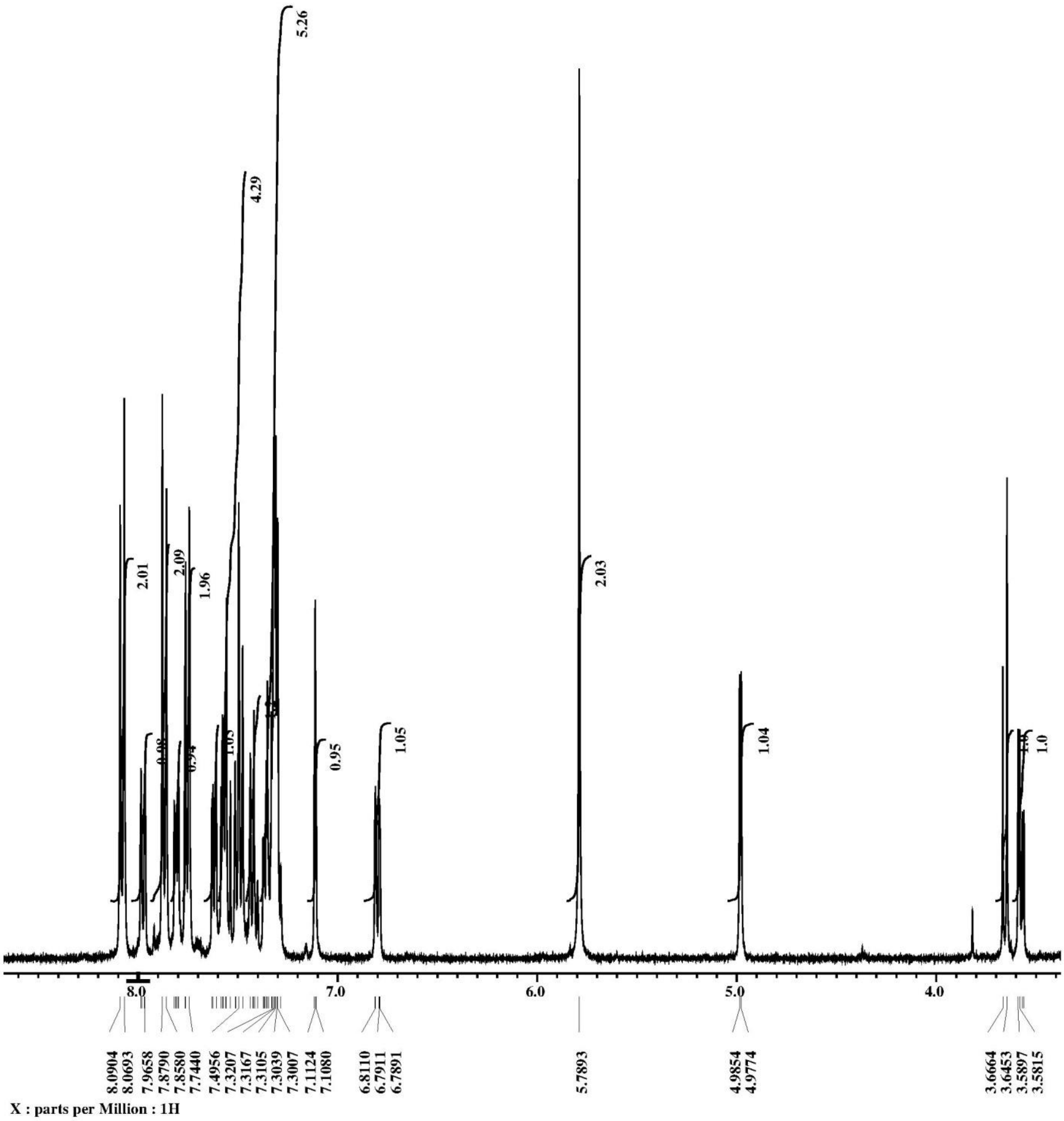

Figure S2. ^{1}H NMR spectrum of NL in DMSO-d$_6$.

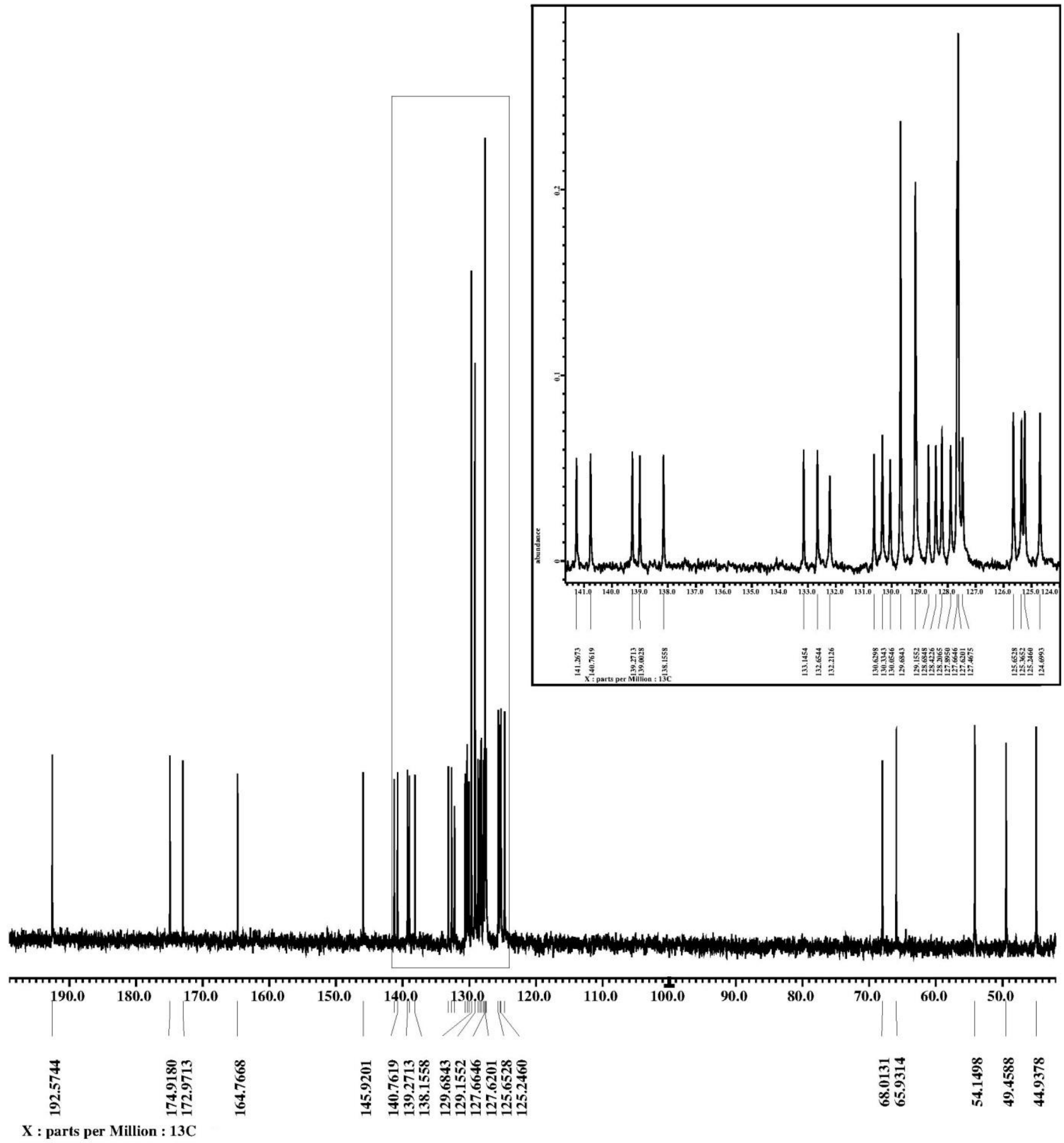

Figure S3. $^{13}C\{^1H\}$ NMR spectrum of NL in DMSO-d_6.

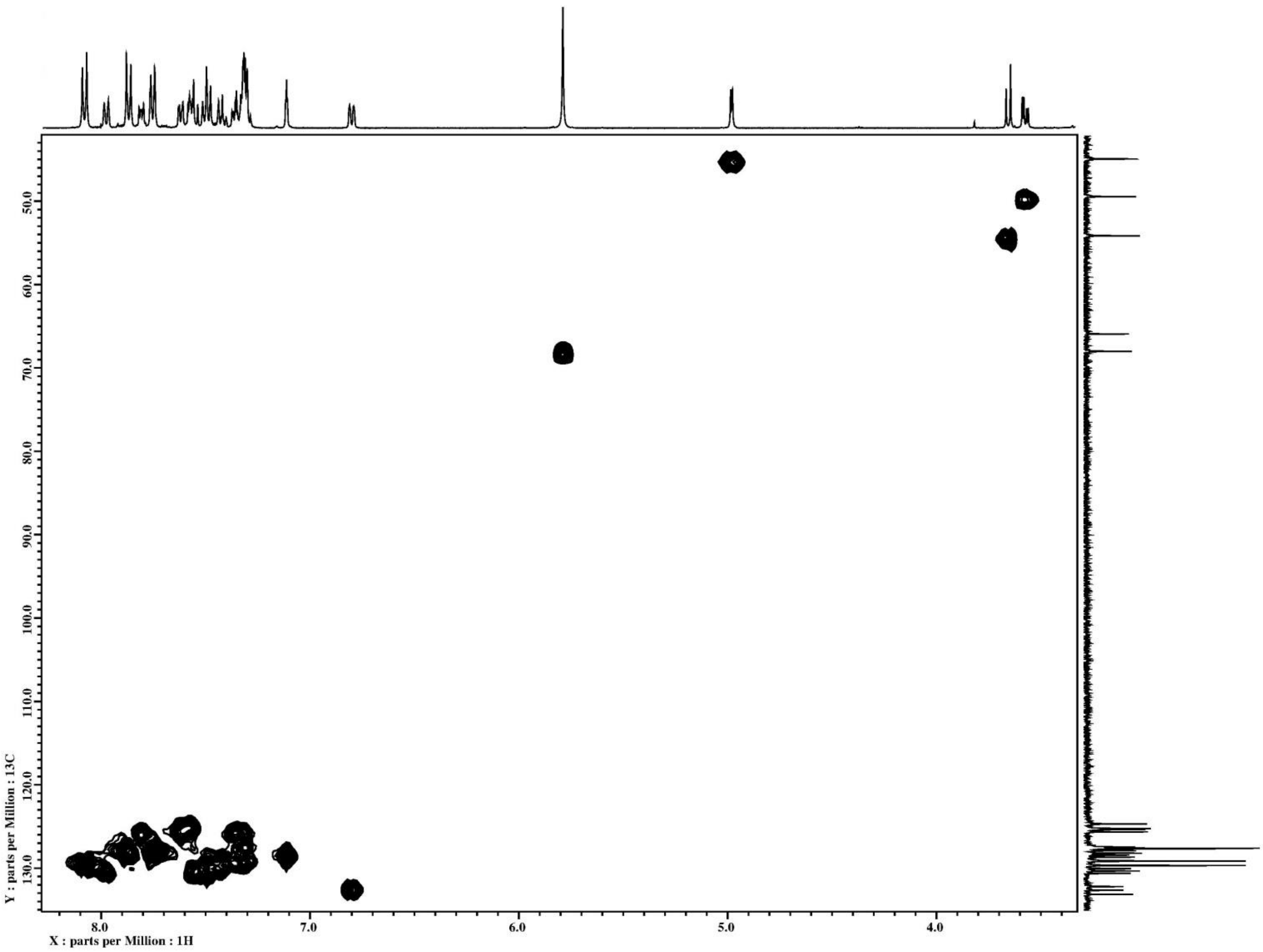

Figure S4. ^{1}H–^{13}C HMQC spectrum of NL in DMSO-d$_6$.

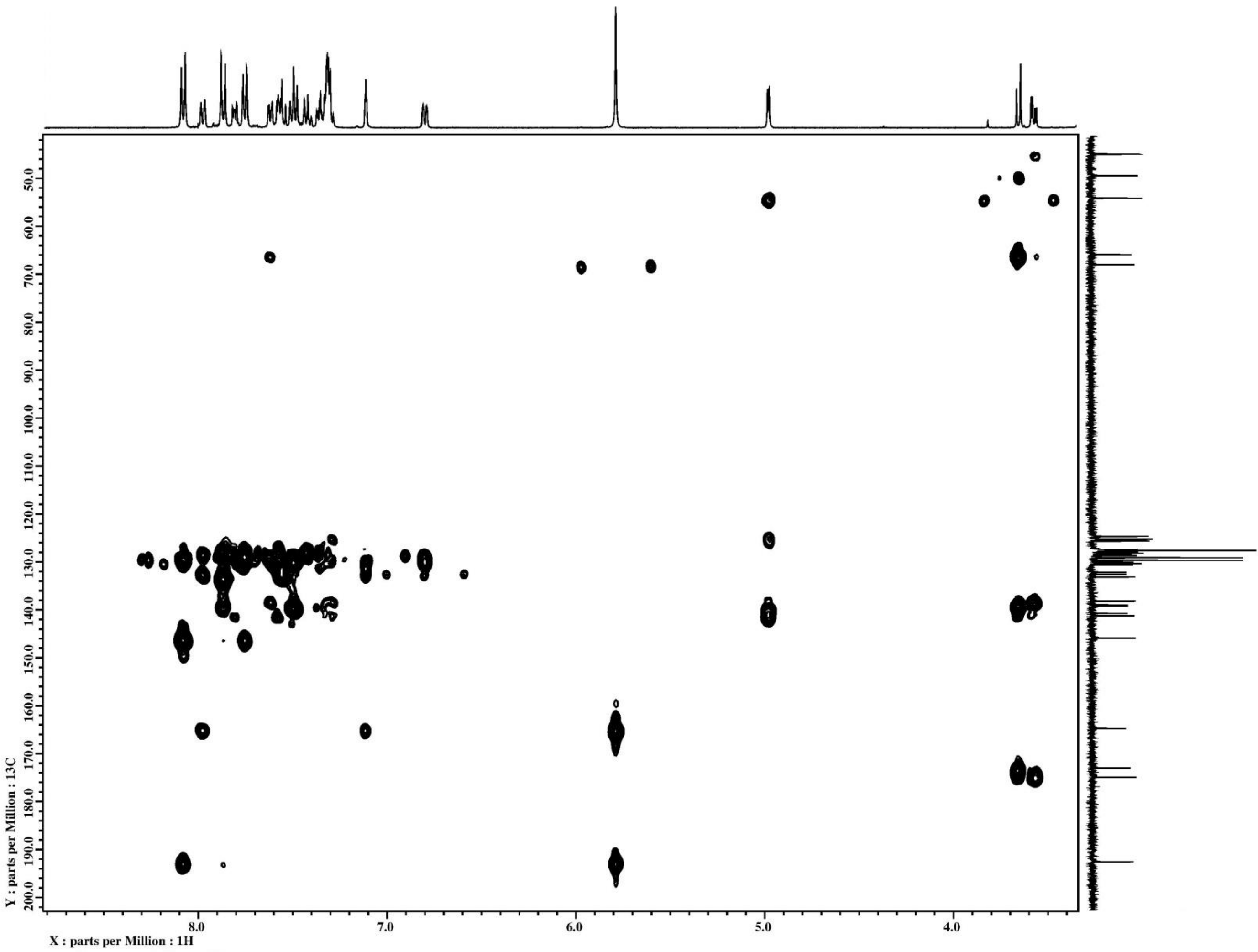

Figure S5. ^{1}H–^{13}C HMBC spectrum of NL in DMSO-d_6.

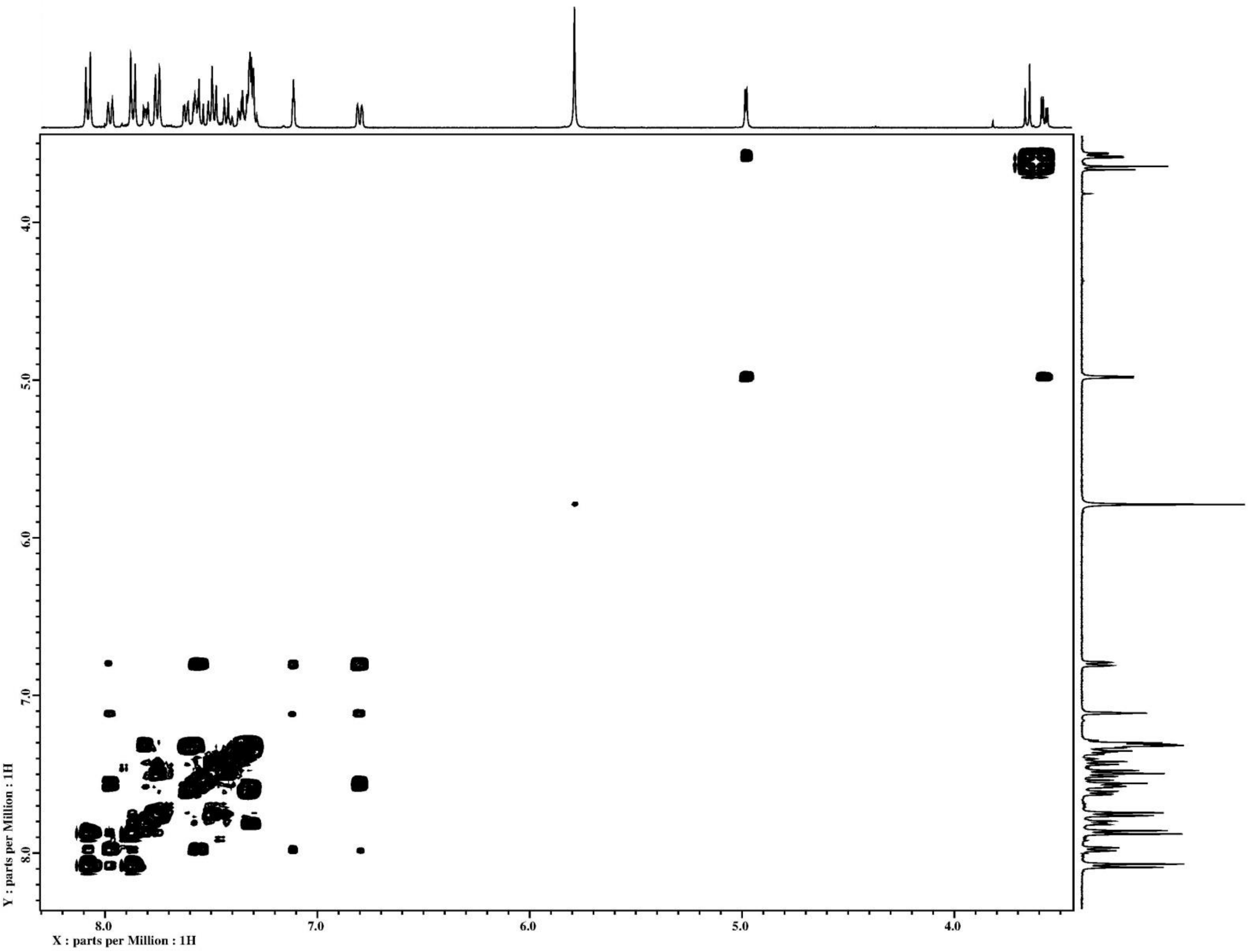

Figure S6. ^1H–^1H dqf-COSY spectrum of NL in DMSO-d_6 (mix time 0.5 s).

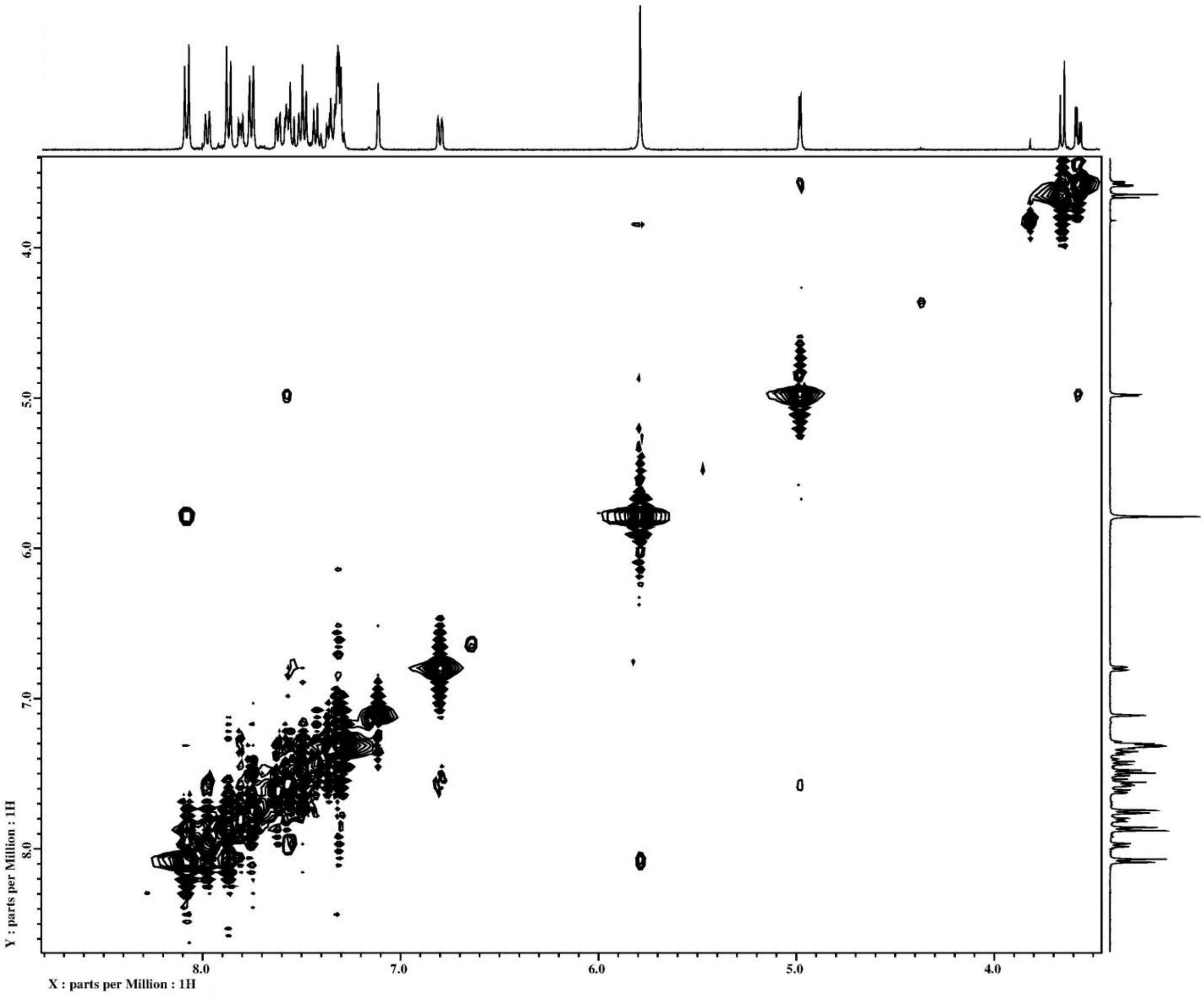

Figure S7. $^{1}H–^{1}H$ NOESY spectrum of NL in DMSO-d_6 (mix time 0.5 s).

Display Report

Analysis Info

Analysis Name	D:\Data\2026\March\24\11299.d	Acquisition Date	24.03.2026 10:59:36
Method	20230815_tune_low_pos.m		
Sample Name		Operator	Bruker Customer
Comment		Instrument / Ser#	micrOTOF 10223

Acquisition Parameter

Source Type	ESI	Ion Polarity	Positive	Set Nebulizer	0.4 Bar
Focus	Not active			Set Dry Heater	180 °C
Scan Begin	50 m/z	Set Capillary	4500 V	Set Dry Gas	4.0 l/min
Scan End	1300 m/z	Set End Plate Offset	-500 V	Set Divert Valve	Waste

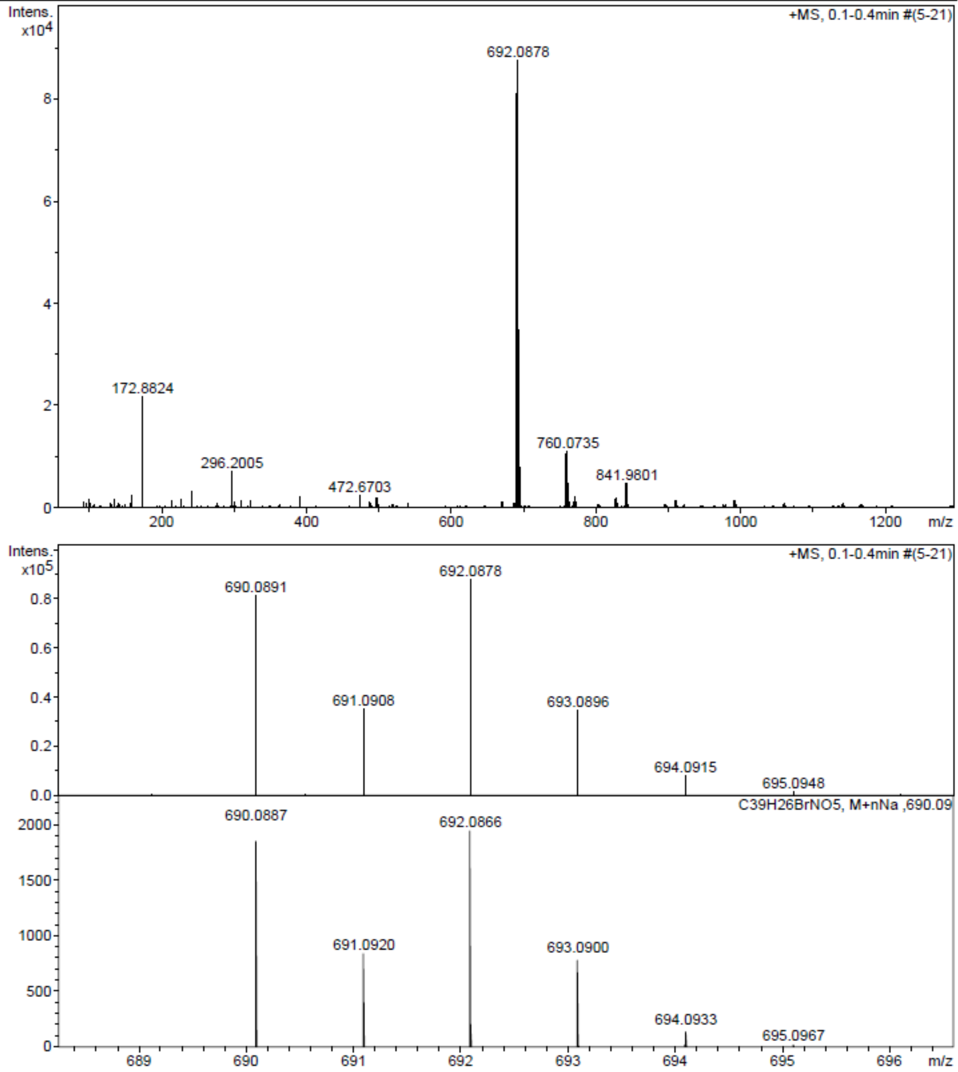

Bruker Compass DataAnalysis 4.0 printed: 24.03.2026 11:30:38 Page 1 of 1

Figure S8. ESI+ mass spectrum of compound NL with a peak at m/z 690.0891 corresponding to [M +Na]+ and a bromine isitopic pattern.

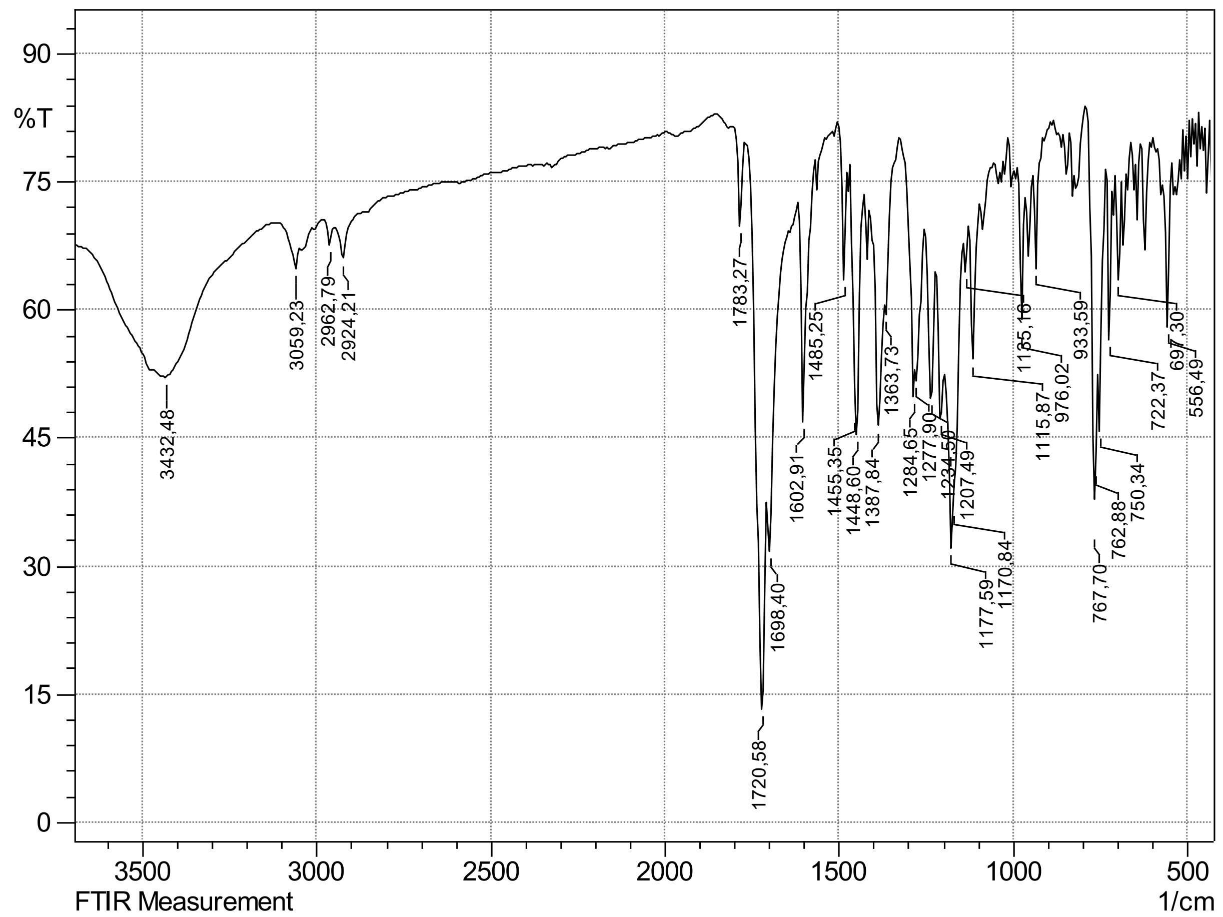

Figure S9. IR spectrum of NL in KBr.

Table S1. Docking results of letermovir (s1) and 72 substances that were taken from the proprietary database with the Tanimoto similarity coefficient ≥ 0.6 to letermovir. The results were obtained by using the Autodock Vina program. S61 is NL

Number of substance	SMILES	Tanimoto score	Affinity
LET (s1)	COC1=C(C=C(C=C1)C(F)(F)F)N2[C@H](C3=C(C(=CC=C3)F)N=C2N4CCN(CC4)C5=CC(=CC=C5)OC)CC(=O)O	1.0	-8.7
s2	Cc1ccc(c(C)c1)N1C(=O)C2C3CCC(C3)C2C1=O	0.62	-6.6
s3	c1ccc2c(c1)c(cc(c1ccc(cc1)Cl)n2)C(=O)OCC(=O)c1ccc(cc1)OC(=O)c1ccco1	0.61	-7.2
s4	CCc1ccc(cc1)O	0.60	-4.5
s5	CCn1c(=O)c2CCCCc2c(c2ccc(c(c2)S(=O)(=O)N2CCC(CC2)C(=O)N)OC)n1	0.60	-7.8
s6	c1ccc2c(c1)C1(c3ccccc3C2(C2C1C(=O)N(c1ccccc1OC(=O)c1ccco1)C2=O)Br)Br	0.60	-8.3
s7	CC1C(=C(C)NC(=C1C(=O)c1ccccc1)C)C(=O)c1ccccc1	0.59	-7.8
s8	Cc1ccc(c(C)c1)NC(=O)c1cc(c2cccs2)nc2c(C)cc(cc12)Br	0.59	-7.7
s9	CCc1ccc(cc1S(=O)(=O)N1CCCCC1C)c1c2CCCCc2c(=O)n(CC)n1	0.59	-7.2
s10	CCc1ccc(cc1S(=O)(=O)Nc1ccc(cc1)C(=O)N)c1c2CCCCc2c(=O)n(C)n1	0.59	-7.4
s11	CC1Nc2ccccc2C(=O)N1c1ccc(cc1)NC=C1C(=O)CC(C)(C)CC1=O	0.59	-7.8
s12	CCOC(=O)C1=C(C)NC2=C(C1c1ccc(cc1)[N+](=O)[O-])C(=O)c1ccccc21	0.59	-6.8
s13	c1cc(C(=O)Oc2ccc(cc2)N2C(=O)C3C4CC(C3C2=O)C(C4Br)Br)oc1	0.59	-6.9
s14	CCn1c(=O)c2CCCCc2c(c2ccc(C)c(c2)S(=O)(=O)Nc2ccccc2C)n1	0.59	-7.8
s15	c1ccc(cc1)C(=O)Oc1ccc(cc1)C(=O)COC(=O)c1ccc(cc1)N1C(=O)c2ccccc2C1=O	0.58	-7.4
s16	Cc1ccc(cc1)CN(C(=O)C)c1c(=O)n(CC(=O)Nc2ccc(c(c2)Cl)F)c2ccccc2n1	0.58	-7.5
s17	CC(=O)N(Cc1ccccc1Cl)c1c(=O)n(CC(=O)Nc2cccc(c2)F)c2ccccc2n1	0.58	-7.8
s18	Cc1ccc(cc1S(=O)(=O)Nc1ccc(cc1)C(=O)N)c1c2CCCCc2c(=O)n(C)n1	0.58	-8.1
s19	CCc1ccc(cc1S(=O)(=O)N1CCC(CC1)C(=O)N)c1c2CCCCc2c(=O)n(C)n1	0.58	-8.0
s20	Cc1ccccc1CNS(=O)(=O)c1cc(ccc1C)c1c2CCCCc2c(=O)n(C)n1	0.57	-7.3
s21	CC(C)C1CCC(C)CC1OC(=O)C1=C(C)NC2=C(C1/C=C/c1ccccc1)C(=O)c1ccccc21	0.57	-8.1
s22	CC(=O)N(Cc1ccc(cc1)F)c1c(=O)n(CC(=O)Nc2c(cccc2F)F)c2ccccc2n1	0.57	-7.4
s23	CCC(C(=O)Nc1ccc(c(c1)OC)OC)n1c(=O)ccc(c2cc(C)ccc2C)n1	0.57	-6.9
s24	CCC(C(=O)Nc1ccc(c(c1)OC)OC)n1c(=O)ccc(c2cc(C)ccc2C)n1	0.57	-7.0
s25	c1ccc2c(c1)C1c3ccccc3C2(C2C1C(=O)N(c1ccccc1C(=O)OCC(=O)c1ccc(cc1)[N+](=O)[O-])C2=O)Br	0.57	-8.2
s26	Cc1ccc(cc1)NC(=O)C(C)n1c(=O)c2CCCCc2c(c2ccc(C)c(c2)S(=O)(=O)NC)n1	0.57	-7.7

s27	CCN(c1cccc(C)c1)C(=O)Cn1c(=O)c2ccccc2c(C)n1	0.57	-6.7
s28	c1ccc(cc1)c1ccc(cc1)C(=O)COC(=O)c1ccccc1N1C(=O)C2C3c4ccccc4C(c4ccccc34)(C2C1=O)Br	0.57	-9.5
s29	c1ccc2c(c1)C1(c3ccccc3C2(C2C1C(=O)N(c1cccc(c1)C(=O)OCC(=O)c1ccc(cc1)OC(=O)c1ccco1)C2=O)Br)Br	0.57	-8.8
s30	c1ccc2c(c1)C(=O)N(Cc1cccnc1)C2=O	0.57	-6.9
s31	Cc1ccccc1NC(=O)C(C)n1c(=O)c2CCCCc2c(c2ccc(C)c(c2)S(=O)(=O)NC)n1	0.57	-7.9
s32	Cc1ccc(cc1S(=O)(=O)NC)c1c2CCCCc2c(=O)n(CC(=O)Nc2ccc(cc2)F)n1	0.57	-7.8
s33	Cc1ccc(c(C)c1)NC(=O)C(CC(=O)c1ccccc1)n1c(C)c(Cc2ccccc2)c(C)n1	0.57	-7.7
s34	CC(C(=O)Nc1cc(c(cc1OC)OC)Cl)n1c(=O)ccc(c2ccccc2)n1	0.57	-6.9
s35	CCC(C(=O)Nc1ccc2c(c1)OCCO2)n1c(=O)ccc(c2ccccc2)n1	0.56	-7.1
s36	Cc1ccc(c(c1)Nc1ccc(c2ccc(C)c(c2)S(=O)(=O)N)nn1)OC	0.56	-6.8
s37	Cc1cccc(c1)NC(=O)C(C)n1c(=O)c2CCCCc2c(c2ccc(C)c(c2)S(=O)(=O)N)n1	0.56	-8.2
s38	Cc1ccc(cc1)NC(=O)C(C)n1c(=O)c2CCCCc2c(c2ccc(C)c(c2)S(=O)(=O)N)n1	0.56	-8.3
s39	c1ccc2c(c1)C1c3ccccc3C2(C2C1C(=O)N(c1ccccc1C(=O)OCC(=O)c1cccc(c1)[N+](=O)[O-])C2=O)Br	0.56	-8.3
s40	COc1ccc(cc1)c1cc(c2ccccc2n1)C(=O)O	0.56	-6.3
s41	Cc1cc(C)c(c(C)c1)NC(=O)Cn1c2ccccc2nc(c1=O)N(Cc1ccc(cc1)F)C(=O)C	0.56	-7.9
s42	c1ccc2c(c1)C1c3ccccc3C2C2C1C(=O)N(Cc1cccnc1)C2=O	0.56	-7.7
s43	CCC(C(=O)Nc1ccc2c(c1)OCO2)n1c(=O)ccc(c2ccccc2)n1	0.56	-7.0
s44	Cc1ccc(C)c(c1)c1c2CCCCc2c(=O)n(CC(=O)Nc2cc(ccc2F)F)n1	0.56	-8.3
s45	CCC(C(=O)Nc1ccc2c(c1)OCCO2)n1c(=O)c2CCCCc2c(c2ccc(C)cc2)n1	0.56	-8.1
s46	Cc1ccccc1NC(=O)C(C)n1c(=O)c2CCCCc2c(c2ccc(C)c(c2)S(=O)(=O)N)n1	0.56	-7.8
s47	CCc1ccccc1NC(=O)Cn1c(=O)c2CCCCc2c(c2ccc(C)c(c2)S(=O)(=O)NC)n1	0.56	-7.7
s48	Cc1ccccc1CN(C(=O)C)c1c(=O)n(C(C)C(=O)N2CCc3ccccc23)c2ccccc2n1	0.56	-8.6
s49	Cc1cccc(c1)NC(=O)C(C)n1c(=O)c2CCCCc2c(c2ccc(C)c(c2)S(=O)(=O)NC)n1	0.56	-8.2
s50	Cc1ccc(cc1)CN(C(=O)C)c1c(=O)n(CC(=O)Nc2c(C)cc(C)cc2C)c2ccccc2n1	0.56	-7.7
s51	CCC(C(=O)Nc1ccc2c(c1)OCO2)n1c(=O)c2CCCCc2c(c2ccc(C)cc2)n1	0.56	-7.9
s52	CC(C(=O)Nc1cc(ccc1OC)OC)n1c(=O)ccc(c2ccccc2)n1	0.56	-6.7
s53	Cc1ccc(c(C)c1)NC(=O)C(C)n1c(=O)c2CCCCc2c(c2ccc(C)c(c2)S(=O)(=O)N)n1	0.56	-8.2
s54	CC1(C)Cc2c(cnn2c2ccccc2)C(=O)/C/1=N\NC(=O)c1ccc(cc1)[N+](=O)[O-]	0.56	-7.8
s55	c1cc(ccc1CC(=O)Oc1ccc(cc1)Br)Cl	0.56	-6.7
s56	Cc1ccc(cc1)NS(=O)(=O)c1cc(ccc1C)c1c2CCCCc2c(=O)n(C)n1	0.56	-7.6
s57	COc1ccc(cc1)NC(=O)c1cccc(c1)N1C(=O)C2C3CCC(C3)C2C1=O	0.56	-7.7
s58	CCCCCCCOC(=O)c1ccc(cc1)N1C(=O)C2C(C1=O)C1(c3ccccc3C2(c2ccccc12)Br)Br	0.56	-7.1

s59	CC(C)COC(=O)c1ccccc1Nc1ccc(cc1[N+](=O)[O-])[N+](=O)[O-]	0.56	-5.9
s60	Cc1cc2c(C)cc(=O)[nH]c2c(c1)S(=O)(=O)N1CCCC1	0.56	-6.1
s61	c1ccc(cc1)c1ccc(cc1)C(=O)COC(=O)c1cccc(c1)N1C(=O)C2C3c4ccccc4C(c4ccccc34)(C2C1=O)Br	0.55	-10.1
s62	CC(C(=O)N1CCc2ccccc12)n1c2ccccc2nc(c1=O)N(Cc1ccc(cc1)F)C(=O)C	0.55	-8.2
s63	CC(C(=O)Nc1ccc(cc1OC)OC)n1c(=O)ccc(c2ccccc2)n1	0.55	-6.8
s64	CCc1ccc(cc1S(=O)(=O)Nc1ccccc1C)c1c2CCCCc2c(=O)n(C)n1	0.55	-7.6
s65	CC(C)(C)C(=O)COC(=O)c1cc(c2cccc(c2)N2C(=O)C3C4CCC(C4)C3C2=O)nc2ccccc12	0.55	-8.5
s66	CCc1ccc(cc1S(=O)(=O)N1CCc2ccccc2C1)c1c2CCCCc2c(=O)n(C)n1	0.55	-8.4
s67	CCn1c(=O)c2CCCCc2c(c2ccc(c(c2)S(=O)(=O)Nc2ccc(C)cc2C)OC)n1	0.55	-7.5
s68	c1ccc2c(c1)C1c3ccccc3C2(C2C1C(=O)N(CC(=O)O)C2=O)Br	0.55	-7.2
s69	c1ccc(cc1)C(=O)COC(=O)c1ccc(cc1)N1C(=O)C2C(C1=O)C1(c3ccccc3C2(c2ccccc12)Br)Br	0.55	-8.3
s70	COc1ccc(cc1)C(=O)COC(=O)CN1C(=O)C2C(C1=O)C1(c3ccccc3C2(c2ccccc12)Br)Br	0.55	-7.4
s71	Cc1ccc(cc1)Oc1ccc(cc1)NC(=O)c1ccccc1Cl	0.55	-7.0
s72	CCCCCOC(=O)c1ccccc1N1C(=O)C2C(C1=O)C1(c3ccccc3C2(c2ccccc12)Br)Br	0.55	-7.1
s73	COC(=O)c1ccccc1N1C(=O)C2C3c4ccccc4C(c4ccccc34)(C2C1=O)Br	0.55	-8.1
